# Development of the Czech Childhood Cancer Information System: Data Analysis and Interactive Visualization

**DOI:** 10.2196/23990

**Published:** 2021-06-29

**Authors:** Denisa Krejčí, Matěj Karolyi, Lucie Pehalová, Jakub Ščavnický, Michaela Zapletalová, Ivana Katinová, Jaroslav Štěrba, Jan Starý, Lenka Šnajdrová, Martin Komenda, Ladislav Dušek

**Affiliations:** 1 Institute of Biostatistics and Analyses Faculty of Medicine Masaryk University Brno Czech Republic; 2 Institute of Health Information and Statistics of the Czech Republic Prague Czech Republic; 3 Facilty of Informatics Masaryk University Brno Czech Republic; 4 Department of Paediatric Oncology Institutions shared with the Faculty Hospital Brno, Faculty of Medicine Brno Czech Republic; 5 Department of Paediatric Haematology and Oncology Charles University and University Hospital Motol Prague Czech Republic

**Keywords:** cancer epidemiology, children, data visualization, software development

## Abstract

**Background:**

The knowledge of cancer burden in the population, its time trends, and the possibility of international comparison is an important starting point for cancer programs. A reliable interactive tool describing cancer epidemiology in children and adolescents has been nonexistent in the Czech Republic until recently.

**Objective:**

The goal of this study is to develop a new web portal entitled the Czech Childhood Cancer Information System (CCCIS), which would provide information on childhood cancer epidemiology in the Czech Republic.

**Methods:**

Data on childhood cancers have been obtained from the Czech National Cancer Registry. These data were validated using the clinical database of childhood cancer patients and subsequently combined with data from the National Register of Hospitalised Patients and with data from death certificates. These validated data were then used to determine the incidence and survival rates of childhood cancer patients aged 0 to 19 years who were diagnosed in the period 1994 to 2016 (N=9435). Data from death certificates were used to monitor long-term mortality trends. The technical solution is based on the robust PHP development Symfony framework, with the PostgreSQL system used to accommodate the data basis.

**Results:**

The web portal has been available for anyone since November 2019, providing basic information for experts (ie, analyses and publications) on individual diagnostic groups of childhood cancers. It involves an interactive tool for analytical reporting, which provides information on the following basic topics in the form of graphs or tables: incidence, mortality, and overall survival. Feedback was obtained and the accuracy of outputs published on the CCCIS portal was verified using the following methods: the validation of the theoretical background and the user testing.

**Conclusions:**

We developed software capable of processing data from multiple sources, which is freely available to all users and makes it possible to carry out automated analyses even for users without mathematical background; a simple selection of a topic to be analyzed is required from the user.

## Introduction

Childhood and adolescent cancers (or *childhood cancers* for short) are classified among rare diseases as their incidence rates are orders of magnitude lower than cancer incidence rates in adults. About 400 new cases of childhood cancers are diagnosed in the Czech Republic each year. Although childhood cancers are rare in terms of absolute numbers, they are the second leading cause of death among children (after injuries).

The web portal SVOD (System for Visualisation of Oncology Data) [1] provides representative epidemiological data on cancer in the Czech Republic. However, it is not entirely convenient for childhood cancers. The main issue is a different classification system of childhood cancers, which takes into consideration differences between childhood and adult cancers; unlike the classification system of adult cancers, which is based on the site of the primary tumor, the classification system of childhood cancers is primarily based on morphology.

For this reason, the Institute of Health Information and Statistics of the Czech Republic (IHIS CR), together with the Institute of Biostatistics and Analyses at the Faculty of Medicine of the Masaryk University (IBA FM MU), the Department of Paediatric Oncology at the University Hospital Brno, and the Department of Paediatric Haematology and Oncology at the University Hospital in Motol, decided to develop a new web portal entitled the Czech Childhood Cancer Information System [2] (CCCIS), which would provide information on childhood cancer epidemiology in the Czech Republic.

The primary objective of the CCCIS portal is to provide comprehensible overviews of epidemiological data on the incidence of childhood cancers in the Czech Republic and mortality and survival data related to cancers in childhood and adolescents. The CCCIS portal also aims to provide relevant information on childhood cancer in the Czech Republic to the international community; this is the reason why the portal is available not only in the Czech language but also in English. The authors of this paper asked the following exploratory questions, which are answered in the Discussion section: how can detailed analytical views—covering incidence, mortality, and survival—be methodically and technically designed and subsequently implemented; is it possible to determine epidemiological trends of selected cancer diagnoses, based on available representative data; and what are the current survival rates in childhood cancer patients in the Czech Republic?

## Methods

### Data Sources

Data on childhood cancers, which are used on the portal, have been obtained from the Czech National Cancer Registry (CNCR), which is administered by IHIS CR [3]. These data were validated using the clinical database of childhood cancer patients [4] and subsequently combined with data from the National Register of Hospitalised Patients [5] and with data from death certificates [6]. These validated data were then used to determine incidence and survival rates of childhood cancer patients aged 0 to 19 years who were diagnosed in the period 1994 to 2016. Data from death certificates were used to monitor long-term mortality trends. Demographic data on the population of interest were obtained from outputs of the Czech Statistical Office [7]. International data sources were used as well: incidence data from the International Incidence of Childhood Cancer [8], mortality data from the European Cancer Information System [9], and survival data from the international comparative study CONCORD-3 [10].

### Classification

Cancers were classified into 12 main groups, according to the International Classification of Childhood Cancer, 3rd Edition (ICCC-3) [11]. All diagnostic groups (I-XII) with behavior 3 (primary malignant tumors) plus diagnoses from groups III (central nervous system [CNS] and miscellaneous intracranial and intraspinal neoplasms) and Xa (intracranial and intraspinal germ cell tumors) with behavior 0/1 (benign neoplasms or those of uncertain or unknown behavior) were considered to be malignant tumors. As regards mortality data, the most common causes of childhood cancer deaths according to the International Statistical Classification of Diseases, Tenth Revision (ICD-10) [12] are shown, namely, the following list of diagnoses: all malignant neoplasms (C00-C97); malignant neoplasms of bone and articular cartilage (C40-C41); malignant neoplasms of connective and soft tissue (C47, C49); malignant neoplasms of brain, spinal cord, and other parts of the CNS (C70-C72); non-Hodgkin lymphoma (C82-C86); and leukemias (C91-C95).

### Analysis of Epidemiological Data

In terms of statistical analyses, the web portal CCCIS focuses on three epidemiological indicators: incidence, mortality, and survival. Incidence is the number of new cases diagnosed in a given period in a given population. The CCCIS portal makes it possible to express the incidence in several different ways. First, absolute numbers show the overall burden of the population with a given disease. Second, crude incidence is the number of new cases arising per 1 million children in a given population. If the population of interest only comprises persons in a given age interval (eg, 10-14 years), we are talking about an age-specific incidence. Third, the age-standardized incidence is the theoretical incidence rate that a given population would have if it had a standard age structure. The portal makes it possible to calculate the European age-standardized incidence rate (ASR-E) [13] and the world age-standardized incidence rate (ASR-W) [14]. Mortality is the number of deaths from a given diagnosis (the so-called cause-specific mortality) occurring in a given period in a given population. As is the case with incidence rates, mortality on the CCCIS portal can also be expressed in absolute numbers, rates per 1 million children, age-specific rates, and age-standardized rates to European or world standard population. Overall survival rates were used to evaluate the patients’ survival, corresponding to the overall monitored survival, regardless of the cause of death. The overall 1-, 2-, 3-, 4-, and 5-year survival was calculated using the life tables method with 1-year intervals, where death from any cause was the event of interest.

### Design and Development

CCCIS is a web portal equipped with an online data browser, which has been developed using the modern and practice-proven Symfony PHP framework in version 3.4 [15]. Using this framework for systematic design, development, and implementation of web applications significantly accelerates individual stages and generally facilitates the applications’ administration and extensibility. It is therefore possible to react relatively quickly to users’ needs and requirements to adapt current functionalities or to add new functionalities. The Twig template engine has been used to create page templates, and the Doctrine Object Relational Mapper has been used to map the objects—both had been released together with the Symfony framework. The data repository has been built on an open-source object-oriented system, PostgreSQL, which is currently routinely used to organize more complex data structures [16]. The main advantages of PostgreSQL include the support of the developer community, the possibility of advanced performance optimization, and a high quality of technical documentation, making it possible to administer the entire database system and the individual databases without problems.

The import itself into the database is performed by automatic scripts, which upload the new database contents. Maintaining the uniformity of the data model and data purity and quality (thoroughly validated by analysts and developers) are essential requirements for a successful import. The dependence on third-party libraries is dealt with by Yarn (front end) and Composer (back end). A large proportion of the portal has been designed to be responsive (ie, displayed content is automatically adjusted depending on the user’s device—desktop, tablet, mobile phone). However, responsiveness is not complete in several parts containing the data browser; the mobile version is not fully supported in this instance because the user interface is far too complex (graphics, filters, analysis settings). The responsive front end has been developed using the Zurb Foundation framework and the jQuery library. The webpack tool has been used to compile the final package of styles and Javascript functions. The interactive data browser requires a special functionality, which is provided by extension components of Javascript libraries; in particular, d3.js, NVD3, and Datatables have been used for interactive data visualizations. We had already applied a similar approach to the development of web applications in the past, namely, in interactive data browsers focused on several issues in Czech health care [17-20].

### Data and Application Security

One of the basic requirements of this project was to secure the entire application, including the data layer. The application has been designed to resist potential third-party attacks and to respond flexibly. The communication between the client and the server takes place in a secure way via the HTTPS protocol. This encrypted transmission is nowadays used as standard, and it is common practice to secure the flow of data in this way. The application itself, built on the Symfony framework, has other security mechanisms that are built into it. Respecting standard implementation approaches ensures that exposure to basic attacks such as cross-site scripting, cross-site request forgery, or various types of injection, especially SQL injection, is avoided.

Server-side protection is provided mainly by a network firewall. Another effective way to detect possible third-party attacks is to set up well-configured resource and traffic monitoring, log errors and accesses from the external internet environment, and alert the administrator to nonstandard events.

Protection of the data itself is another necessary requirement to be met. The underlying data, which are used by the portal for rendering visualizations, are cleaned of all personal and sensitive information on patients and their hospital stays. Therefore, it is impossible to directly connect the records to a specific patient. The data are securely stored in a database that is accessible only from predefined locations (these are always part of an internal network) and to a limited set of users whose permissions are restricted to certain data operations. The data are always sent to the client side in an aggregated form, as required for the final visualization. It is important to perform the aggregation operation before sending so that the data cannot be broken down into individual rows at the client side. At the same time, with potentially small numbers of records, the result of the analysis is not sent, and the user is notified of this fact. Thus, the identification of a specific person is effectively prevented.

### Validation and User Testing

Feedback was obtained and the accuracy of outputs published on the CCCIS portal was verified using the following methods, which are generally suitable to identify potential shortcomings not only in terms of contents but also in terms of design of the user interface and control elements.

The *validation of theoretical background*, which describes the basic terms, the methodology of cancer classification, and static analytical reports, was performed internally (ie, in cooperation with the analytical team and a group of senior doctors who are specialists in childhood cancers and have many years of experience with the methodology of childhood cancer classification). At the same time, all three sections of the interactive data browser were thoroughly checked; complex analytical views of incidence, mortality, and survival rates according to user settings were extensively tested.

*User testing* involved simple instructions to go through individual sections of the portal and to provide subjective feedback as regards the overall visual style, control elements, and user-friendliness.

The outputs from both assessments were extensively discussed by the team of authors, and selected suggestions, which fit in with the overall concept of the portal, were subsequently implemented.

## Results

### Basic Description of the CCCIS Portal

The web portal CCCIS [2] is a stand-alone online presentation, which has been freely available on the internet since November 2019, without the necessity of user authentication. The portal is allowed to be indexed and therefore to be found by standard search engines. Users can access the published contents via a web browser, and all communication takes place via the HTTPS protocol (ie, in a secured and encrypted manner). The CCCIS portal is divided into several sections:

- The *Introduction* section provides basic information about the portal objectives and contents. Participating institutions and the team of authors are introduced. This section also describes the source of data that have been used for statistics and for interactive data views. News related to childhood cancers in the Czech Republic are also involved.
- The *Methodology* section describes how childhood cancers are classified according to the international classification system.
- The *Statistics* section contains an overview of information and descriptive attributes on provided views of the data set. The section is divided into incidence, mortality, and survival. Static analyses are also available for download; however, this feature is only available in the Czech language.
- The *Interactive data views* section provides graphical outputs, which make it possible for users to go through available data sets in an interactive manner. All data sets are regularly updated, based on data from the CNCR and data from a clinical database. Like the Statistics section, the Interactive data views section is divided into incidence, mortality, and survival subsections. This section of the portal is described in more detail in the next section of this paper.
- The *Publications* section provides a list of articles published in research journals and a list of conference papers.

### Introduction of the CCCIS Browser

The interactive browser is the principal component of the portal, containing predefined analytical tools that make it possible for the user to look into epidemiological data from different points of view, both in graphical and tabular representation. From the user’s viewpoint, this is how the interactive browser is used:

- Selection of the main module (incidence, mortality, survival)
- Selection of analysis type
- Selection of the analyzed group of patients, setting the analysis outputs

Selection of the main module is the first step to begin with any analysis. The principal epidemiological analyses, the so-called modules, cover the following topics: incidence, mortality, and survival. After selecting the main module, the user needs to select the required type of analysis, which means analysis by year of diagnosis, by sex, by cancer type, by age and cancer type combined (this option is only available in the incidence and mortality modules), or by international comparison. Individual types of analyses can be selected in the upper part of the screen. After selecting the required analysis type, an analytical window is displayed, showing the results with basic settings. These settings can be further adjusted on two levels.

#### Options for the Analyzed Group of Patients

Options for the analyzed group of patients can be selected using the following filters:

- Diagnosis (or cancer type): selection of diagnosis by ICCC-3 (in the incidence and survival modules) or by ICD-10 (in the mortality module)
- Sex: the entire population of children, boys only, or girls only
- Age: selection of age categories 0 to 19 years, 0 to 14 years, <1 year, 1 to 4 years, 5 to 9 years, 10 to 14 years, or 15 to 19 years (in the incidence and mortality modules), or 0 to 19 years, 0 to 14 years, or 15 to 19 years (in the survival module)
- Period/years: a scrollbar can be used in the incidence and mortality modules to select individual years or a span of years (currently between 1994 and 2016); as for the survival module, only the predefined periods 1999 to 2004, 2005 to 2010, and 2011 to 2016 can be selected

These filters can be combined, and a detailed view of selected topics can be obtained in this way. Unsuitable or illogical variables in the context of the selected analysis are inactive (gray). The *reset filters* button can be used to restore the original analysis settings.

#### Detailed Settings of the Analysis Output

Depending on the selected analysis, the software offers a suitable computational method such as absolute numbers; annual numbers; percentages; rate per 1 million children; ASR-E; ASR-W; and 1-, 2-, 3-, 4-, or 5-year overall survival rate. The toggle switch *Group years?* in the Incidence and Mortality modules makes it possible to visualize data for individual years (the *off* position) or for years grouped together (the *on* position). In the *International comparison* analysis, this toggle switch is always in the *on* position, making it possible to compare data from the Czech Republic with data from other European countries.

The primary output of the interactive tool is a graph displayed in the center of the working window, including the description of applied filters and the data source. Apart from this graphical output, results can also be displayed in the form of a data table. Graphical outputs can be downloaded as images, whereas tabular outputs can be copied, printed, or downloaded as *.csv or *.xlsx files. Short reports describing the main epidemiological indicators have been written to provide basic overviews of selected diagnoses.

**Figure 1:**
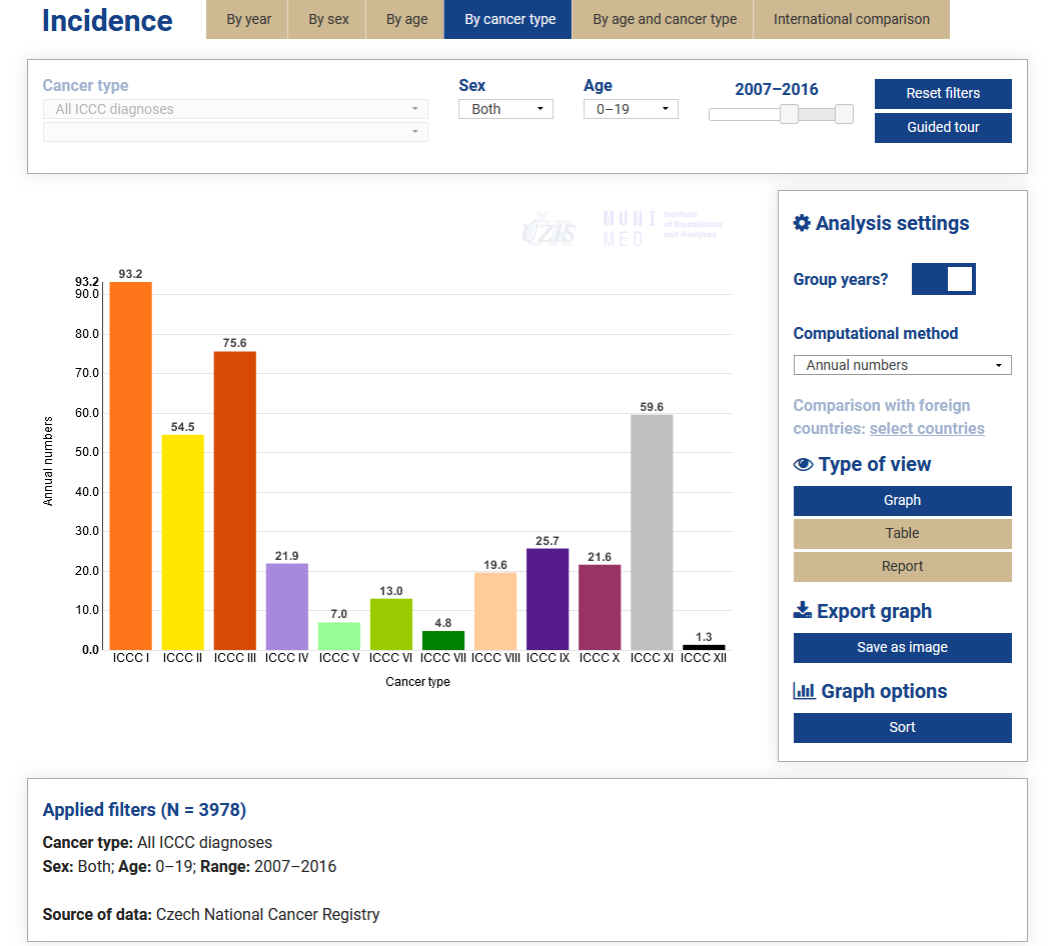
Czech Childhood Cancer Information System Interactive data views, incidence by cancer type. ICCC: International Classification of Childhood Cancer.

As an illustration, Figure 1 shows the Incidence module, namely, the analysis by cancer type, with the toggle switch *Group years?* being in the *on* position, *annual numbers* selected as the computational method, the time filter set to the period 2007 to 2016, and the graph sorted in descending order. We can see that leukemias (ICCC I) are the most common childhood cancers (ie, those diagnosed in patients aged 0 to 19 years), followed by CNS tumors (ICCC III), other malignant epithelial neoplasms, and malignant melanomas (ICCC XI) and lymphomas (ICCC II).

**Figure 2:**
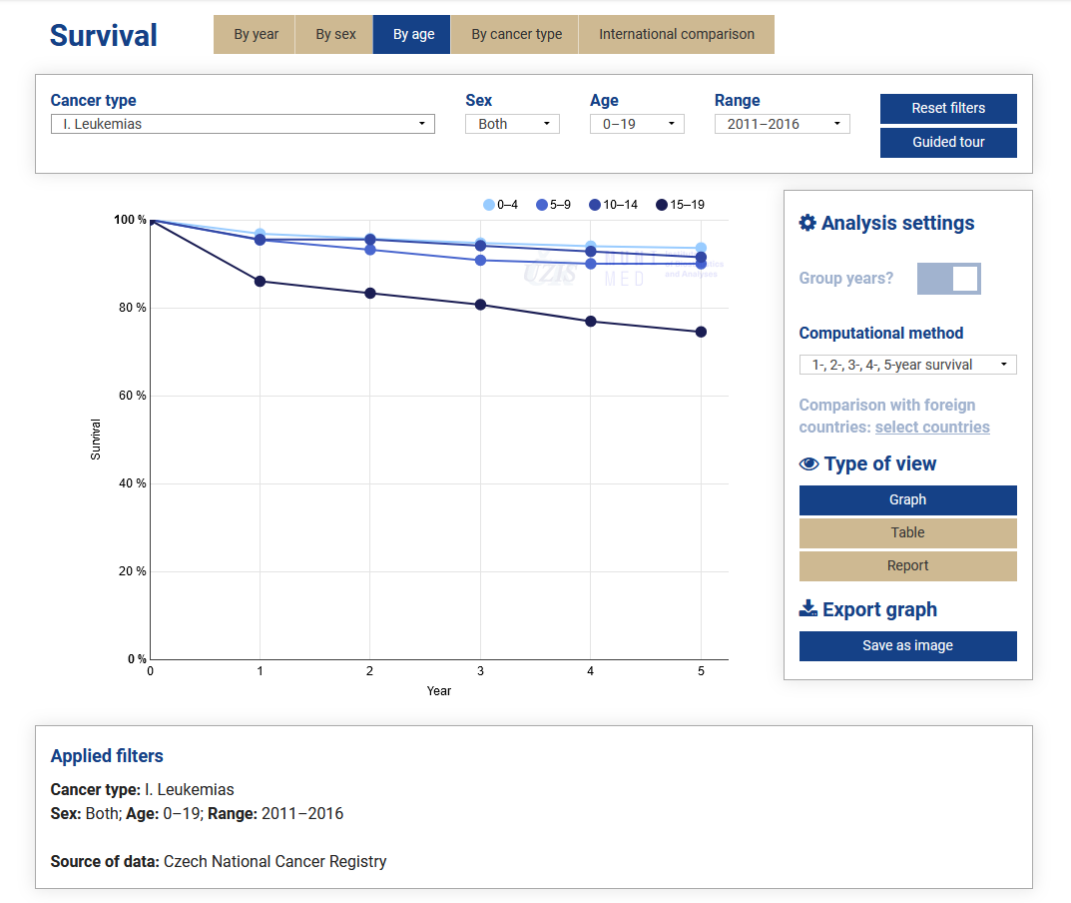
Czech Childhood Cancer Information System Interactive data views, survival by age.

For illustrative purposes, the Survival module is described (see Figure 2), namely, the analysis by age, with *Leukemias* selected as the cancer type and 1-, 2-, 3-, 4-, and 5-year survival selected as the computational method. We can see that survival rates in the period from 2011 to 2016 differ significantly: patients aged 15 to 19 years have markedly lower 5-year overall survival rates than younger patients.

### Benefit for Users

The software is capable of processing data from multiple sources, is freely available to all users, and makes it possible to carry out automated analyses even for users without mathematical background; a simple selection of a topic to be analyzed is required from the user. This online available software tool makes it therefore possible for anyone to display long-term trends of childhood cancer incidence, mortality, and survival, and to compare data from the Czech Republic to those from abroad. All analytical outputs are displayed in both graphical and tabular outputs. The aggregation of data over time periods is another indisputable advantage of the interactive browser.

### Evaluation of the CCCIS Portal

The validation of the static contents was carried out as an internal review of static texts and analytical reports. The comments were of rather formal character, aiming to unify the published information from the linguistic and visual points of view. The CCCIS portal was also assessed by experts, namely, by two senior doctors dealing with childhood cancer patients on a daily basis, who were asked to follow a given scenario using the CCCIS portal (full description of this scenario including feedback is available at [21]).

## Discussion

### Principal Results

The main findings of this study are summarized in the following discussion in the form of answers to three exploratory questions, which were asked in the Introduction section.

How can detailed analytical views—covering incidence, mortality, and survival—be methodically and technically designed and subsequently implemented? The basic methodical concept of the CCCIS portal lies in the division of the presented contents into three parts (theoretical background, interactive data views, analytical reports), which make up a compact set of information comprehensible to experts in the field and health care professionals. Clear and appropriate definitions of basic terms, together with the description of the International Classification of Childhood Cancer, explain relevant issues to users. The interactive browser represents a unique dynamic tool that makes is possible to set different views of available data sets, including the type of view (graph, table, report) and subsequent download. Analytical reports provide a summary overview of childhood cancer epidemiology in the Czech Republic and detailed reports for individual ICCC groups. The technical solution itself is based on the robust PHP development Symfony framework, with the PostgreSQL system used to accommodate the data basis.

Is it possible to determine epidemiological trends of selected cancer diagnoses based on available representative data? The interactive browse makes epidemiological data available through predefined analytical tools. Users can look into epidemiological indicators—incidence, mortality, and survival of selected cancer diagnosis—by selecting the main module (incidence, mortality, or survival), followed by the selection of the required type of analysis (by year of diagnosis, sex, age, cancer type, age and cancer type combined, and international comparison), and the selection of the patient group to be involved in the analysis. Absolute numbers; annual numbers; rates per 1 million children; age-standardized rates to European or world standard population; and the overall 1-, 2-, 3-, 4-, and 5-year survival can be displayed, depending on the selected analysis.

What are the current survival rates in childhood cancer patients in the Czech Republic? The overall 1-, 2-, 3-, 4-, and 5-year survival rates for 12 main ICCC groups can be obtained using the main *Survival* module of the interactive browser, after selecting the type of analysis and possibly by being more specific about the group of patients to be involved in the analysis. The highest 5-year survival in the period 2011 to 2016 was observed in retinoblastoma (ICCC V), and in other malignant epithelial neoplasms and malignant melanomas (ICCC XI). By contrast, the lowest 5-year survival rates were observed in soft tissue and other extraosseous sarcomas (ICCC IX) and in hepatic tumors (ICCC VII).

### Future Visions

The CCCIS portal will be further developed within the joint workplace of the IHIS CR and IBA FM MU. Comments and suggestions provided by users themselves will play an important role in this process. Specific questions will be formulated in cooperation with the expert society, probably requiring additional analytical outputs.

### Comparison With Other Works

Publicly available portals describing childhood cancer epidemiology in other European countries, namely, in Ireland [22], Switzerland [23], and the United Kingdom [24], have become our motivation. The Irish web portal provides an interactive analytical reporting for incidence; the Swiss and the UK websites are static but provide information not only on incidence but also on mortality and survival of childhood cancer patients. The new CCCIS portal combines the approaches previously mentioned and thus enables interactive analytical reporting of incidence, mortality, and survival.

### Conclusions

The CCCIS portal is the result of a long-term cooperation of a state organization directly coming under the Ministry of Health of the Czech Republic and selected specialized workplaces in the academic sphere. Doctors, representatives of a department administering the National Health Information System, data analysts, systems analysts, graphic designers, and developers have worked together to create a platform that makes accessible valuable and interesting views of available data in a user-friendly form. The web portal is available for anyone at [2], providing basic information for experts (ie, analyses and publications) on individual diagnostic groups of childhood cancers. It involves an interactive tool for analytical reporting, which provides information on the following basic topics in the form of graphs or tables: incidence, mortality, and overall survival.

## Abbreviations

ASR-E: European age-standardized incidence rate
ASR-W: world age-standardized incidence rate
CCCIS: Czech Childhood Cancer Information System
CNCR: Czech National Cancer Registry
CNS: central nervous system
IBA FM MU: Institute of Biostatistics and Analyses at the Faculty of Medicine of the Masaryk University
ICCC-3: International Classification of Childhood Cancer, 3rd Edition
ICD-10: International Statistical Classification of Diseases, Tenth Revision
IHIS CR: Institute of Health Information and Statistics of the Czech Republic
SVOD: System for Visualisation of Oncology Data

## References

[1] Dusek L, Muzik J, Kubasek M, Koptikova J, Zaloudik J, Vyzula R (2005). Epidemiology of Malignant Tumours in the Czech Republic.

[2] Krejci D, Scavnicky J, Zapletalova M, Svobodova I, Karolyi M, Muzik J, Jarkovsky J, Klimes D, Loula Z, Komenda M, Sterba J, Stary J, Dusek L (2018). Czech Childhood Cancer Information System.

[3] Czech National Cancer Registry. National Health Information System.

[4] Bajciova V, Dusek L, Janotova I, Kabickova E, Kepak T, Klimes D, Kodytkova D, Luks A, Stary J, Smelhaus V, Sterba J, Vavra V, Vrzalova A Czech National Informational and Educational Portal.

[5] National Register of Hospitalised Patients. National Health Information System.

[6] Death certificate system. National Health Information System.

[7] Czech demographic handbook - 2016. Czech Statistical Office.

[8] Steliarova-Foucher E, Colombet M, Ries LAG, Hesseling P, Moreno F, Shin HY, Stiller CA (2017). Results. International Incidence of Childhood Cancer 3.

[9] Incidence and mortality historical data. ECIS - European Cancer Information System.

[10] Allemani C, Matsuda T, Di Carlo V, Harewood R, Matz M, Nikšić M, Bonaventure A, Valkov M, Johnson CJ, Estève J, Ogunbiyi OJ, Azevedo E Silva G, Chen W, Eser S, Engholm G, Stiller CA, Monnereau A, Woods RR, Visser O, Lim GH, Aitken J, Weir HK, Coleman MP, CONCORD Working Group (2018). Global surveillance of trends in cancer survival 2000-14 (CONCORD-3): analysis of individual records for 37 513 025 patients diagnosed with one of 18 cancers from 322 population-based registries in 71 countries. Lancet.

[11] Steliarova-Foucher E, Stiller C, Lacour B, Kaatsch P (2005). International Classification of Childhood Cancer, third edition. Cancer.

[12] World Health Organization (2016). International Statistical Classification of Diseases and Related Health Problems - 10th Revision, edition 5.

[13] Eurostat (2013). Revision of the European Standard: Population Report of Eurostat's Task Force: 2013 edition.

[14] Doll R, Payne P, Waterhouse J (1966). Cancer Incidence in Five Continents: A Technical Report.

[15] Symfony documentation. Symfony.

[16] Juba S, Volkov A (2019). Learning PostgreSQL 11: A Beginner's Guide to Building High-Performance PostgreSQL Database Solutions. 3rd edition.

[17] Karolyi M, Komenda M, Janoušová R, Vita M, Schwarz D (2017). Finding overlapping terms in medical and health care curriculum using text mining methods: rehabilitation representation - a proof of concept. MEFANET J.

[18] Dušek L, Mužík J, Karolyi M, Šalko M, Malúšková D, Komenda M, Hřebíček J, Denzer R, Schimak G, Pitner T (2017). A pilot interactive data viewer for cancer screening. Environmental Software Systems. Computer Science for Environmental Protection 12th IFIP WG 5.11 International Symposium, ISESS 2017, Zadar, Croatia, May 10-12, 2017, Proceedings.

[19] Karolyi M, Krejčí J, Ščavnický J, Vyškovský R, Komenda M (2019). Tools for development of interactive web-based maps: application in healthcare.

[20] Komenda M, Bulhart V, Karolyi M, Jarkovsky J, Muzik J, Majek O, Snajdrova L, Ruzickova P, Razova J, Prymula R, Mackova B, Brezovsky P, Marounek J, Cerny V, Dusek L (2020). Complex Reporting of the COVID-19 Epidemic in the Czech Republic: Use of an Interactive Web-Based App in Practice. J Med Internet Res.

[21] Evaluation of the CCCIS portal. Czech Childhood Cancer Information System.

[22] National Cancer Registry Ireland.

[23] Childhood Cancer Registry.

[24] Cancer Research UK.

